# Structural remodeling and oligomerization of human cathelicidin on membranes suggest fibril-like structures as active species

**DOI:** 10.1038/s41598-017-14206-1

**Published:** 2017-11-13

**Authors:** Enea Sancho-Vaello, Patrice François, Eve-Julie Bonetti, Hauke Lilie, Sebastian Finger, Fernando Gil-Ortiz, David Gil-Carton, Kornelius Zeth

**Affiliations:** 1 Unidad de Biofisica, Centro Mixto Consejo Superior de Investigaciones Científicas-Universidad del País Vasco/Euskal Herriko Unibertsitatea (CSIC,UPV/EHU), Barrio Sarriena s/n, Leioa, Bizkaia Spain; 2 Genomic Research Laboratory, Department of Medical Specialities, Geneva University Hospitals, University of Geneva, Genève, Switzerland; 3 Institute of Chemistry, Martin-Luther-University Halle-Wittenberg, von-Danckelmann-Platz 4, D-06120 Halle (Saale), Germany; 4 CELLS-ALBA Synchrotron Light Source, 08290 Barcelona, Spain; 5 Structural Biology Unit, CIC bioGUNE, Parque Tecnológico de Bizkaia Edificio 800, 48160 Derio, Spain; 6 Roskilde University, Department of Science and Environment, Universitetsvej 1, 4000 Roskilde, Denmark

## Abstract

Antimicrobial peptides as part of the mammalian innate immune system target and remove major bacterial pathogens, often through irreversible damage of their cellular membranes. To explore the mechanism by which the important cathelicidin peptide LL-37 of the human innate immune system interacts with membranes, we performed biochemical, biophysical and structural studies. The crystal structure of LL-37 displays dimers of anti-parallel helices and the formation of amphipathic surfaces. Peptide-detergent interactions introduce remodeling of this structure after occupation of defined hydrophobic sites at the dimer interface. Furthermore, hydrophobic nests are shaped between dimer structures providing another scaffold enclosing detergents. Both scaffolds underline the potential of LL-37 to form defined peptide-lipid complexes *in vivo*. After adopting the activated peptide conformation LL-37 can polymerize and selectively extract bacterial lipids whereby the membrane is destabilized. The supramolecular fibril-like architectures formed in crystals can be reproduced in a peptide-lipid system after nanogold-labelled LL-37 interacted with lipid vesicles as followed by electron microscopy. We suggest that these supramolecular structures represent the LL-37-membrane active state. Collectively, our study provides new insights into the fascinating plasticity of LL-37 demonstrated at atomic resolution and opens the venue for LL-37-based molecules as novel antibiotics.

## Introduction

Antimicrobial peptides (AMPs) are produced by virtually every organism. They are responsible for the broad-spectrum antimicrobial activity of an organism against fungi, bacteria and viruses. AMPs such as human cathelicidin (LL-37) constantly protect the human body from microbes^1–3^. In contrast to small molecule antibiotics, many AMPs (including LL-37) specifically target bacterial membranes known as ‘the Achilles heel of bacterial cells’^2^. In spite of the wealth of data collected on LL-37, the mechanism by which LL-37 interacts with bacteria to remodel their membranes and kill them has remained largely unknown. However with a greater understanding of the LL-37 structure and its membrane-targeting mechanism, structure-based manipulation of this molecule to increase its effectiveness against multi-resistant bacteria is feasible and should attract greater interest for clinical development^4^.

Models for AMP-membrane interactions are summarized by the following three distinct pathways^2,5,6^. The barrel stave model describes the membrane induced assembly of amphipathic peptides into oligomeric transmembrane channels^7^. The toroidal model describes the delineation of a pore architecture formed by peptide channels laterally stabilized via electrostatic lipid head group interactions^8,9^. Finally, the carpet model describes severe membrane perturbation after the release of peptide-lipid complexes in a process similar to that seen in detergent induced membrane destruction^6^. All of the above processes lead, by a variable extent, to the formation of holes in membranes. When applied to bacterial cytoplasmic membranes. These should lead to the breakdown of the transmembrane potential and cell death^5^. All of the above processes lead, by a variable extent, to the formation of holes in membranes when applied to bacterial cytoplasmic membranes.

LL-37 is produced as a precursor protein in human cells (e.g. leukocytes or neutrophils) and processed during its secretion to the skin, lungs, or reproductive tract. The active peptide carries a surplus of positive charge, which is attributed to interactions with the LPS or LTA of the bacterial cell wall, and negatively charged lipids of *in vivo* cytoplasmic membranes^10,11^. Structural analysis of the full-length peptide in SDS or DPC micelles was previously carried out by NMR yielding deviating monomeric structures^12^. Biochemical and biophysical studies indicated the oligomerization of LL-37 under increased salt and at higher peptide concentrations along with an increase in helicity^13^. Activity of the peptide against *E. coli* was enhanced in the presence of anions such as carbonates or phosphates^13^. Studies using various truncated peptide variants showed a correlation between sequence and activity, implicating that peptides covering the central and C-terminal part of the peptide display the highest antibiotic activity^14^. Recently, an extended activity screen of LL-37-derived sequences including length and positional variations led to the development of a molecule termed OP-145 with strong antibacterial and low hemolytic activity. This peptide is currently in clinical trials at stage II and is used as a possible treatment of chronic middle ear infections^10,15,16^.

Because the structure, function, and membrane-interacting mechanism of the natural antibiotic LL-37 was essentially unknown, we started to investigate this mechanism in detail. We determined its atomic structure in solution and the putative membrane interaction states. Together the structures in the absence and presence of detergents revealed discrete detergent binding sites at the dimer interface indicating the presence of *in vivo* lipid binding sites. Further binding sites between dimers induce supramolecular fiber-like oligomerization. The linear architecture of peptide dimers was confirmed by electron micrographs of nanogold-linked peptide samples applied to lipid vesicles. The structural information that was collected implies that fibril-like structures could represent the active form of the peptide adopted on natural membranes during the course of killing bacteria. The structures presented here may further serve as a valid basis for the structure-based design of novel LL-37 peptide-derived antibiotics.

## Results

### The structure of LL-37 displays an anti-parallel dimer

**Figure 1:**
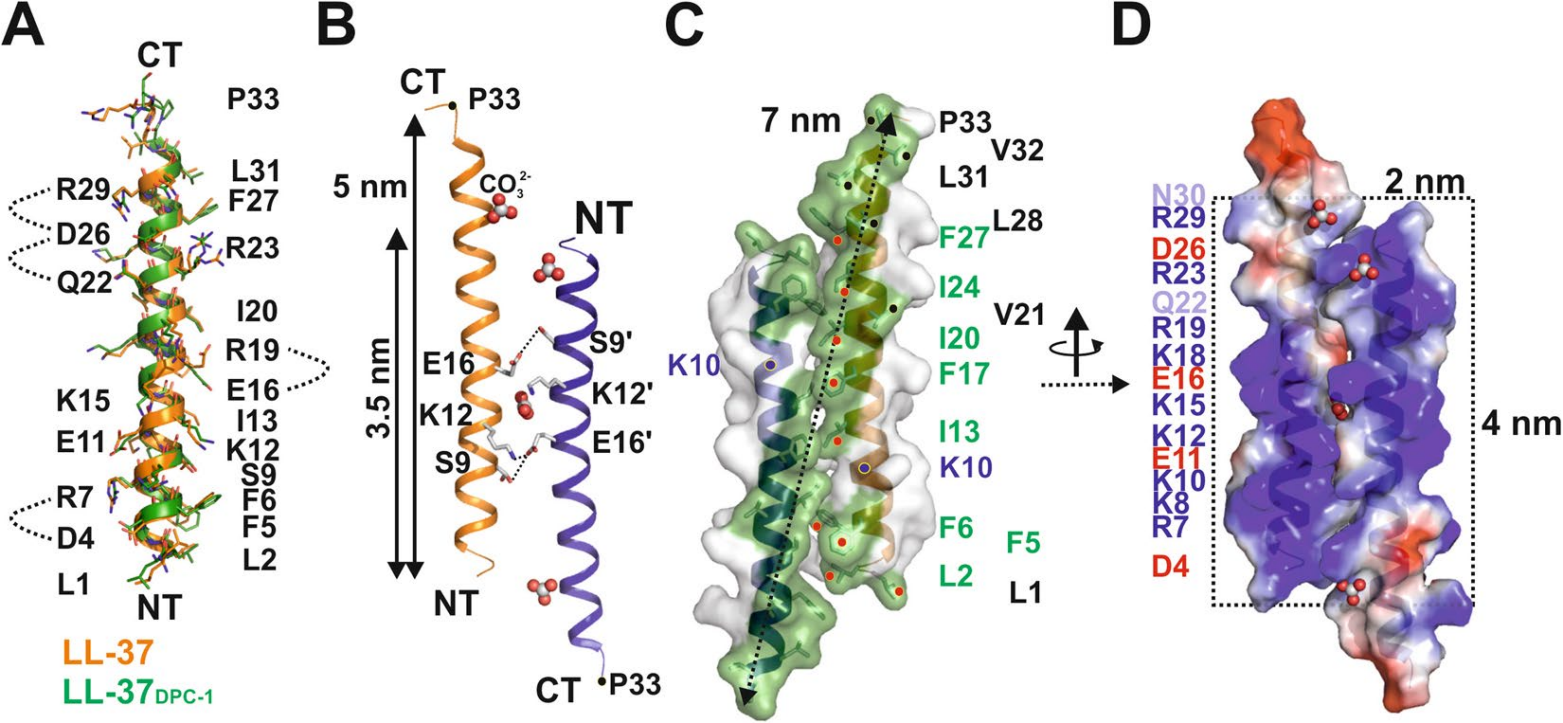
The structure of dimeric LL-37 in solution shows a strongly amphipathic pattern. (**A**) Superposition of LL-37_DPC-1_ (in green) onto LL-37_2_ (in orange) in ribbon and stick representation with all residues and termini (NT and CT) labeled. **(B)** Dimeric and α-helical LL-37_2_ extends to ~5 with an intermolecular interface of 3.5 nm. Hydrophilic residues interacting with each other are marked in stick representation and labelled according to their sequence. **(C)** Surface representation of LL-37_2_, with the hydrophobic belt highlighted stretching over ~7 nm from N- to C’-terminus. Residues partially involved in dimer formation are marked by green numbers and residues extending the hydrophobic belt are marked by black numbers. **(D)** Electrostatic potential of LL-37_2_ mapped on the molecular surface; the blue color represents positive surface potential while the red color signals the negative surface potential distribution.

LL-37 is an extensively studied peptide with particular relevance in human innate immunity^17,18^. To investigate its role in bacterial killing an in-depth investigation of conformational states was initiated based on crystal structures in the presence and absence of detergents. Our first crystals yielded a monomeric structure at atomic resolution (termed LL-37_DPC-1_) obtained under conditions comprising artificially high MPD concentrations (70% MPD; see Fig. 1A). The second structure (termed LL-37_2_) obtained in the absence of detergents displays an anti-parallel dimer formed by two α-helices without supercoiling (see Fig. 1B). The monomers are identical to LL-37_DPC-1_ with each amphipathic helix extending to ~5 nm. Intramolecular stabilization of the monomer is provided by backbone H-bonds and salt bridges (formed between Asp4 and Arg7, Glu16 and Arg19 and Asp26 and Arg29, Gln22 and Asp26; see Fig. 1A). Helices are shifted relative to their termini (N- relative to C-) by approximately two turns, yielding a dimer interface extending to 3.5 nm (see Fig. 1B). The intermolecular interface of the dimer covers the area of 670 Å^2^ (17% of the monomeric surface and residues Leu1 to Phe27), and a biological interface score of ~0.9 as estimated by PISA^19^. Three hydrophilic residues, Ser9, Lys12, and Glu16, form hydrophilic contacts at the dimer interface. In addition, an extended hydrophobic core of residues Leu2, Phe5, Phe6, Ile13, Phe17, Ile20, Ile24, and Phe27 (see Fig. 1C) contributes to the high structural stability with a melting point of 75 degrees estimated by FTIR (see Fig.S1A). Together with the C-terminal hydrophobic residues a 7 nm long exposed hydrophobic surface stretch with a central 4 × 2 nm rectangular area is generated (see Fig. 1C). Interestingly, one discontinuity within this stretch also predicted by the helical wheel model appears at Lys10 (see Figs 1C, S1B and S3A). The opposite side of the dimer is dominated by 20 positive and 8 negative charges which are mostly distributed over the central 4 × 2 nm area of the surface, resulting in a net charge of +12. This high positive charge density of 3.5 charges per nm^2^ is in the same range of lipid head group charges in biological membranes distributed per nm^2^ (see Fig. 1D).

### Detergents induce remodeling of the structure and distinct peptide-detergent interactions are formed

**Figure 2:**
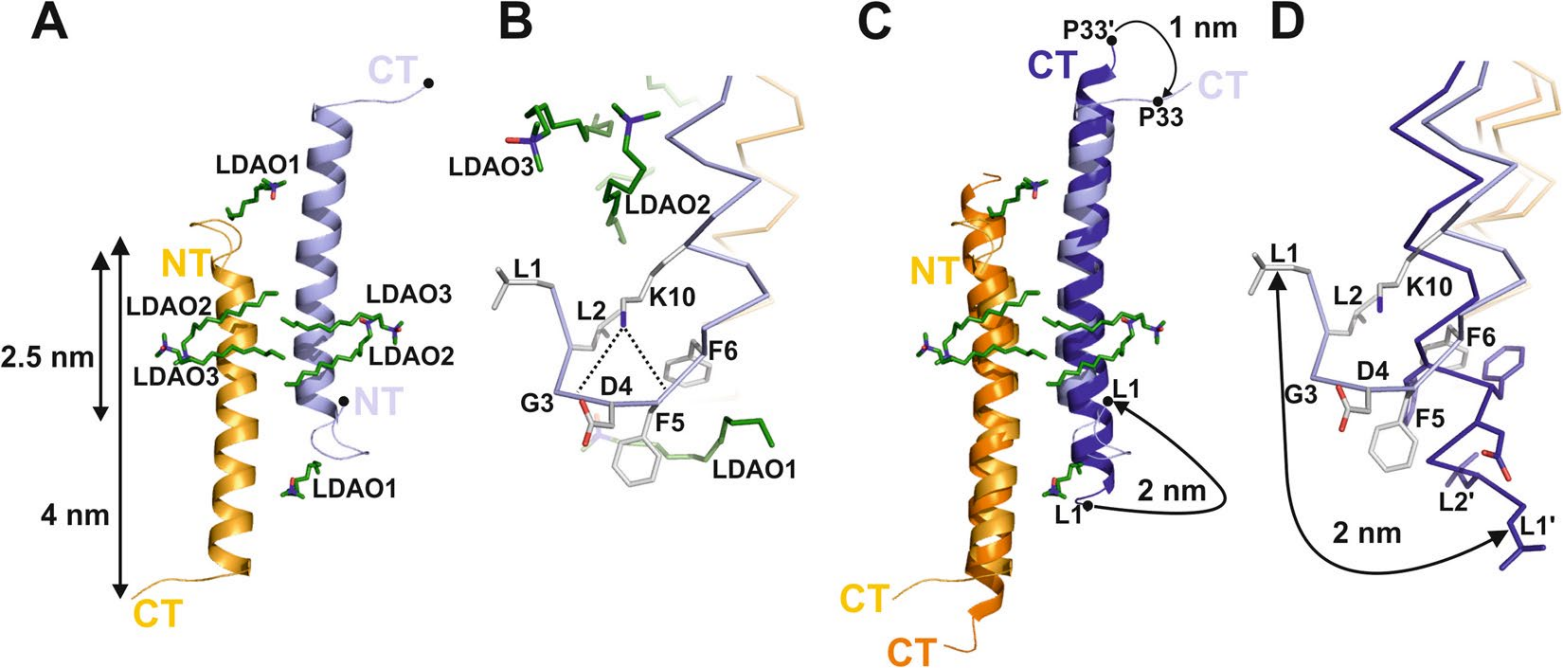
Detergents induce conformational changes leading to supramolecular pattern formation. (**A**) Structure of LL-37 crystallized in the presence of LDAO. The terminal LDAO1 is captured by N- and C-terminal residues (see also Fig. 2B), while LDAO2 and LDAO3 attach to the hydrophobic cavities at the molecular centre. **(B)** Close up view on the N-terminal part of LL-37_LDAO-2_: Phe5 and Phe6 stabilize LDAO1 interactions and Lys10 forms two H-bonds with the backbone carboxyl atoms of Gly3 and Phe5. **(C**,**D)** Superposition of LL-37_2_ and LL-37_LDAO-2_ using the color code established in Figs 1B and 2A, indicating the significant movement of the N-terminus by ~2 nm between the two Leu1 positions (**(D)** is a close up of **(C)**).

**Figure 3:**
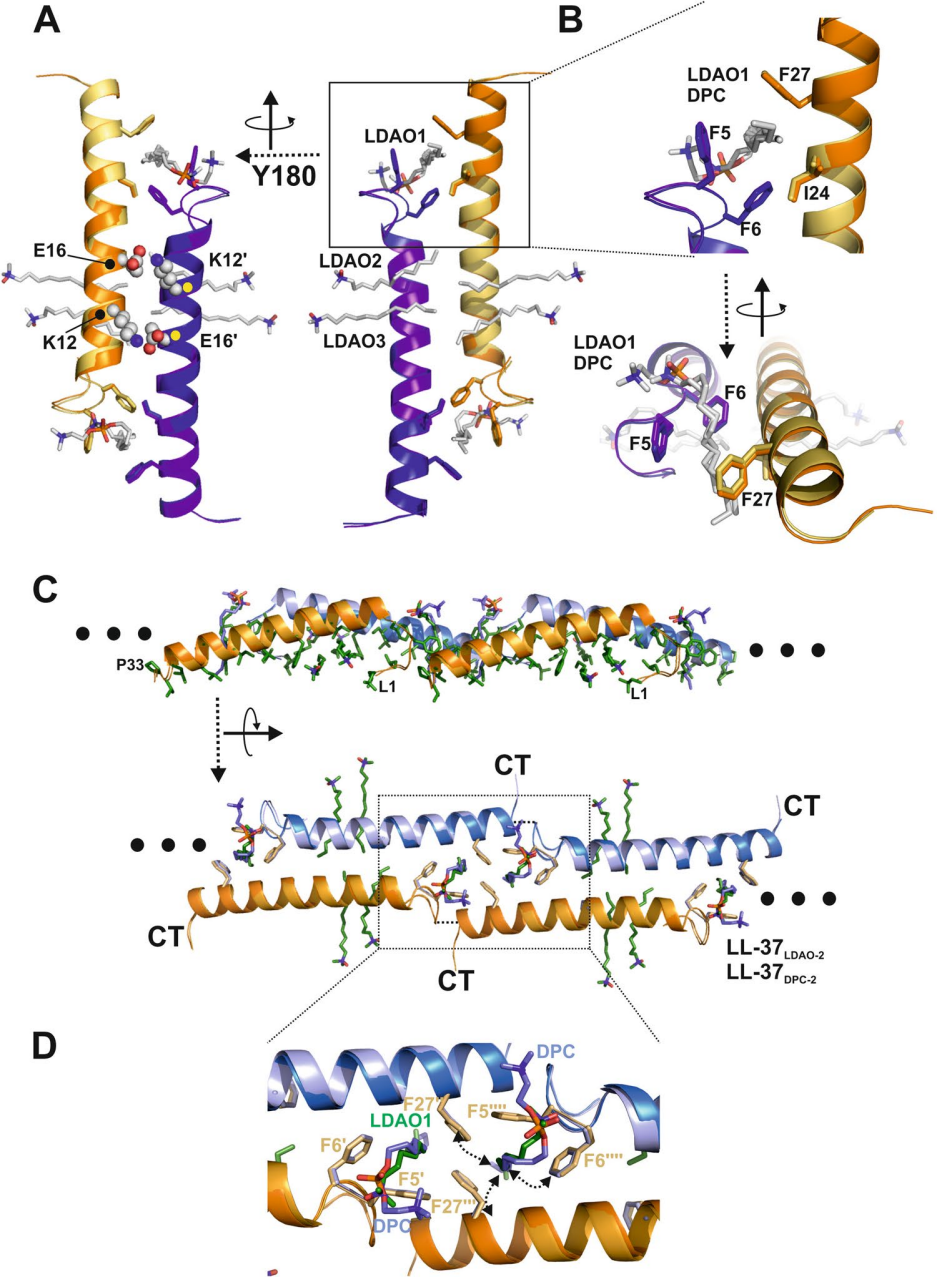
Structural analysis of LL-37 suggests oligomerization and Fiber formation in detergents. **(A)** Positions of detergents in the LL-37_LDAO-2_ and LL-37_DPC-2_ structures. **(B)** Close up view on the LDAO1 and DPC binding sites at the N-terminus. **(C)** Tetramers based on the LL-37_LDAO-2_ structure (light colors) superimpose with low r.m.s.d. on LL-37_DPC-2_ (dark colors) **(D)** Close-up view of the tetramer interface: the residues forming a hydrophobic nest-like structure are encapsulating two detergent molecules labeled by LDAO1 and DPC.

To unravel the membrane-induced conformation of LL-37, we crystallized the peptide in the presence of DPC and LDAO. We obtained two co-crystal structures, both of which are dimeric with anti-parallel helix orientations, containing two DPC (LL-37_DPC-2_) or six LDAO molecules (LL-37_LDAO-2_), respectively (see Fig. 2, Fig. S3 and Table I). The two structures superimpose with very low r.m.s.d of 0.4 Å (for 67 Cα-atoms). However, the structural deviation to LL-37_2_ is significant (2.2 Å for 57 aligned Cα atoms) and only the core region can be reasonably aligned (see Figs 2C,D and S3B). Due to the structural remodeling of the N-terminus in LL-37_LDAO-2_, each monomer extends to only 4 nm and the dimer interface is reduced corresponding to 435 Å^2^ (residues Ser9 to Ile24; see Fig. 2A). The primary structural transition we noticed occurred at the N-terminus (residues Leu1 to Arg7) which translates into a random coil conformation (see Figs 2A–D and S3A and S3B). This transition leads to the exposure of Phe5 and Phe6 for hydrophobic interactions with the detergent alkyl chains (see Fig. 2B and D). Together with the highly conserved residues Ile24 and Phe27 of the second monomer they form a hydrophobic scaffold for the terminal detergent molecules (see LDAO1 in Figs 3A,B and S3). The N-terminal peptide conformation is further stabilized by Lys10 which forms two H-bonds with backbone carbonyls of Gly3 and Phe5 (see Fig. 2B and Fig. S1). The Lys10 residue which is an outlier in the theoretical helix wheel model and LL-37_2_ now appears at a shielded location. There are additional conformational changes accompanying binding of the detergent in the peptide center: e.g. Phe17/Phe17’ adopts a different side chain conformation, and together with Leu1/Leu1’ and Ile13/Ile13’ creates a hydrophobic scaffold for binding four detergent molecules in close vicinity (see Figs 2 and S3A). Interestingly, the four central detergents LDAO2/LDAO2’ and LDAO3/LDAO3’ are oriented such that the two alkyl-chains closely resemble phospholipid tails (see Fig. 2A–D and model S3C for superposition of PE onto LDAO molecules). Also the C-terminus experiences a conformational transition in response to detergent binding via the conserved residues Ile24 and Phe27 (see Figs 2 and S2) whereby the total extension of the monomeric helix is further decreased (see Figs 2 and S3).

### Formation of supramolecular fiber-like assemblies via hydrophobic nest structures

When we analyzed the crystallographic environment of LL-37_LDAO-2_ and LL-37_DPC-2_ (LL-37_LDAO-2_ with one monomer in the AU; LL-37_DPC-2_ with two monomers in the AU)_,_ we noticed a ‘head-to-tail’ arrangement between the peptide dimers forming tetramers and the higher fiber-like structure oligomers. These fiber-like structures were observed in both lattice types (LL-37_LDAO-2_ and LL-37_DPC-2_) although the crystallographic environment does not correspond to each other (see Figs 3, S4A and Table S1). The ‘head-to-tail’ interfaces are formed primarily by the hydrophobic residues Phe5, Phe6 and Phe27, all of which are strongly conserved within the family of cathelicidins (see Figs 3 and S2). These aromatic residues from adjacent chains form nest-like hydrophobic scaffold to embed two detergent molecules per nest (Phe5’, Phe6’, Phe27”, and Phe27”’) from three neighboring monomers (see Figs 3 and S4). Due to the repetitive architecture of these tetramers, the fiber-like structure is formed resulting in a hydrophobic belt with central and terminal detergent interactions. Assuming the detergent positions resemble lipid positions *in vivo*, two lipids per monomeric LL-37 could be extracted from a membrane and incorporated into the nests.

### Gold-labelled LL-37 applied to lipid vesicles shows fiber-like structures corresponding to crystal structures

**Figure 4:**
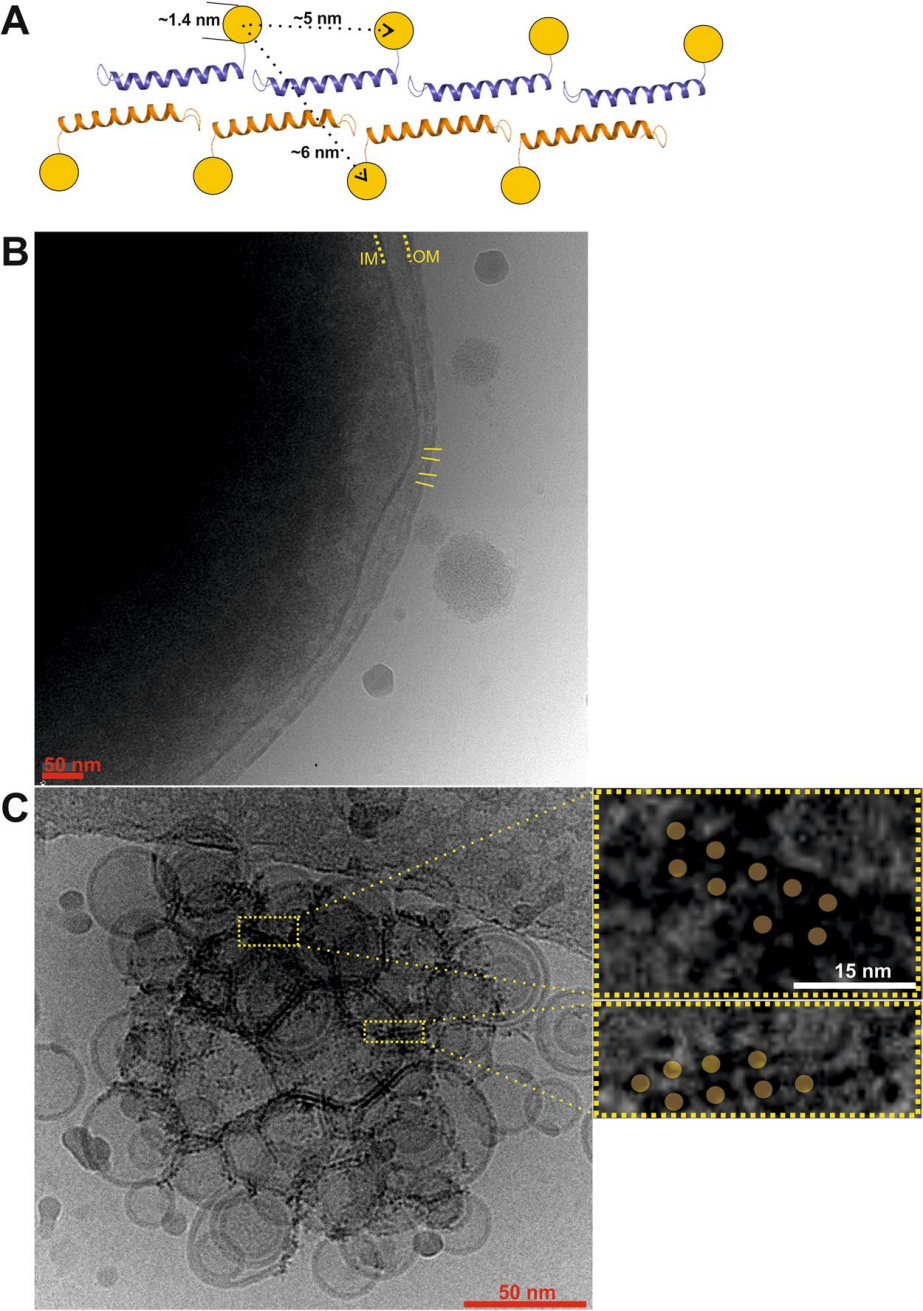
Oligomerization of nanogold labeled LL-37 on lipid vesicles. **(A)** Model of the nanogold-labelled peptide (attached to a S37C mutant) based on the supramolecular architecture observed in the crystal lattice. **(B)** *E. coli* cells incubated with the gold-labelled peptide show the formation of hole-like structures in the outer membrane (OM). **(C)** Vesicles were incubated with nanogold-labelled LL-37 and the contrast of the gold label is visible on vesicles and at vesicle interfaces. On the right, two close-up views of the electron density highlighting the fiber-like structures are shown.

To verify that fiber formation observed in the crystal lattice can also occur on membranes, we labeled the C-terminus of a S37C mutant with nanogold particles 1.4 nm in diameter for electron microscopy studies. If the crystallographic fibers can also be formed on vesicles under the labelling conditions, two repetitive and parallel rows of gold labels should be visible. In these rows the distance between two nanoparticles in each fiber direction is ~5 nm, while the distance vertical to the fiber is ~6 nm (see Fig. 4A). To test if the labelled peptide is visible and active in the context of bacterial cells, *E. coli* was incubated with the purified and labelled peptide and examined by cryo-electron microscopy. The images show a clear perforation of the outer but not of the inner membrane thereby providing evidence for peptide activity; however, nanogold particles are not visible (see Fig. 4B). Therefore, we chose a simplified system for our following investigation and tested if gold-labelled LL-37 would assemble on small unilamellar vesicles. Vesicles were prepared from a mixture of DOPC:DOPG (3:7) and incubated with the peptide at a ratio of 1:250 (peptide:lipid) for 15 minutes. The vesicles showed no significant membrane perturbation as in *E. coli* but small, up to 40 nm long, fiber-like structures of electron-dense spots representing the nanodots were visible on vesicles. The fiber directions follow the curved surface of the vesicles and most of the small fibers show a parallel alignment of dots at a defined distance of about 5–6 nm (see Fig. 4C).

### Lipid head groups determine LL-37 affinity

**Figure 5:**
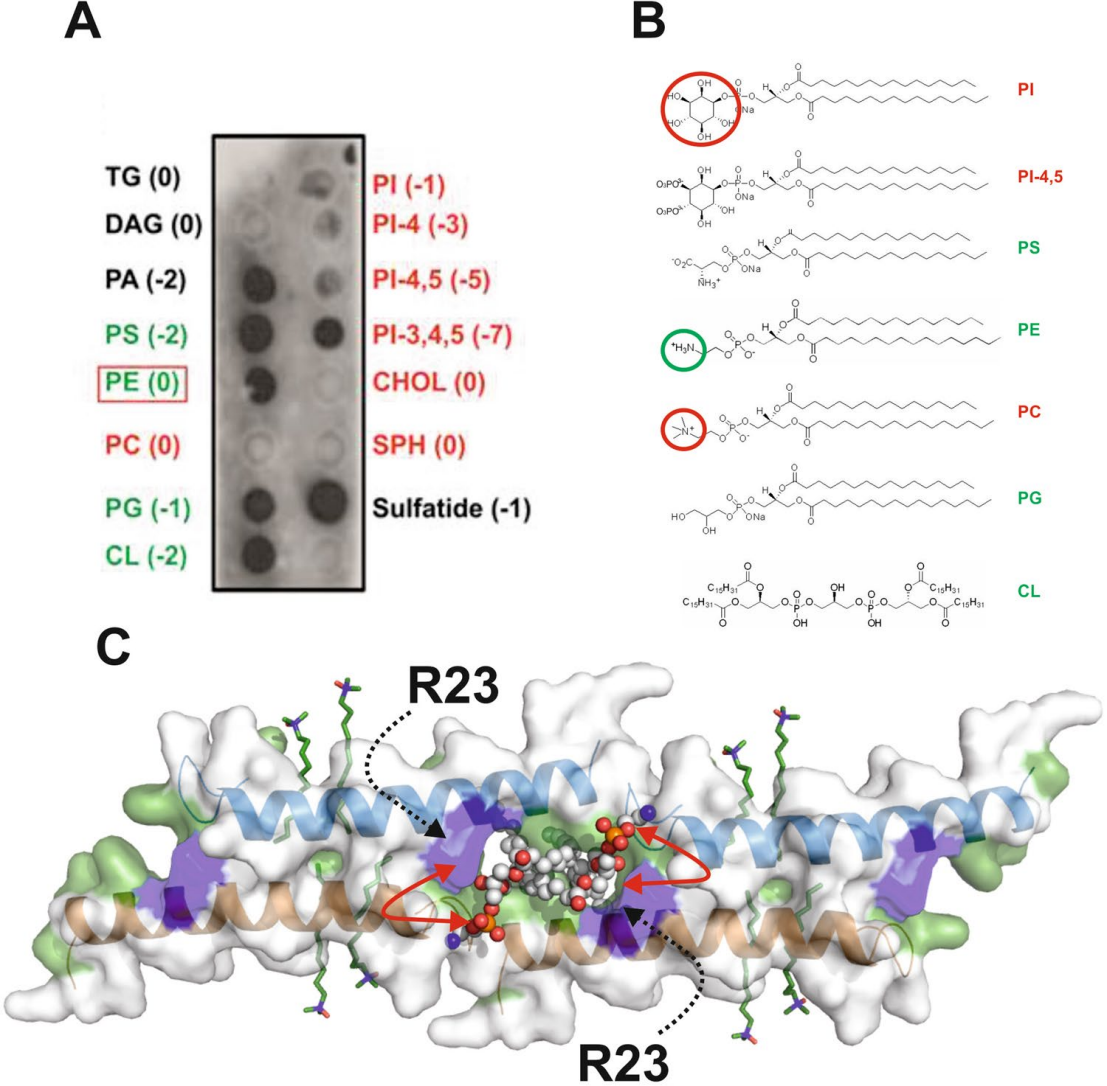
Lipid interactions of LL-37 show clear affinity towards eukaryotic lipid types. **(A)** Interaction between LL-37 and lipids of bacterial (marked in green) or eukaryotic (marked in red) membranes spotted on a nitrocellulose membrane. Antibodies against LL-37 indicate strong interaction between PA, PS, PE, PG, CL, and sulfatide. Of the eukaryotic lipids, only PI-3, 4, 5 are recognized. **(B)** Chemical structures of the lipids tested. **(C)** Model of the nest the structure, with two PE lipids placed onto detergent positions. Head group positions implicate neighborhood to Arg23, which, together with space limiting factors, may be important for lipid selection.

To understand the unique property of LL-37 to selectively act on pro- but not eukaryotic membranes we qualitatively assessed these interactions using lipid spot overlay techniques followed by peptide specific antibody labelling. Bacterial cytoplasmic membranes are primarily composed of phosphatidyl-ethanolamine (PE), phosphatidyl-glycerol (PG), phosphatidyl-serine (PS) and cardiolipin (CL) (for chemical structures see Fig. 5B). Consequently, strong interactions between LL-37 and PS (charge -2), phosphatidic-acid (charge -2), PE (charge 0), PG (charge -1) and CL (charge -2) were observed (see Fig. 5A). Notably, although phosphatidyl-choline (PC) and PE have the same net charge and similar structures, LL-37 does not bind to PC. In essence, the head group charge appears to play a role in these interactions it clearly is not the only determinant as demonstrated by the negatively charged phosphatidyl-inositol (PI - charge -1) and its phosphorylated derivatives PI-4, PI-4,5, PI-3,4,7 (see Fig. 5A and B). PI, PI-4, and PI-4,5 carry negative net charges but they do not interact with LL-37 (see Fig. 5A and B). To understand these characteristics on a structural level we analyzed the terminal detergent-binding mode (see Figs 2, 3 and 5C). In a model where we manually placed PE onto DPC, it is tempting to speculate that the space provided by the hydrophobic nest is sufficient to accommodate two lipid molecules carrying small head groups (see Fig. 5C). We found that the highly conserved Arg23 was in the vicinity of the putative lipid head group and therefore could be of importance for the selection of the positively charged groups (see Fig. S2). However with the space limitation due to the nest structure, a second molecular ruler criterion restricting the head group shape comes into play (see Fig. 5C and Fig. S4B). Testing if Arg23 was essential for activity we engineered a R23A mutant and showed that, while the secondary structure was maintained, the antimicrobial activity against *S. aureus* was lost and the MIC against *E. coli* was increased about tenfold indicating the important influence of this residue for activity (see Fig. S5).

### Biochemical studies of oligomeric LL-37

**Figure 6:**
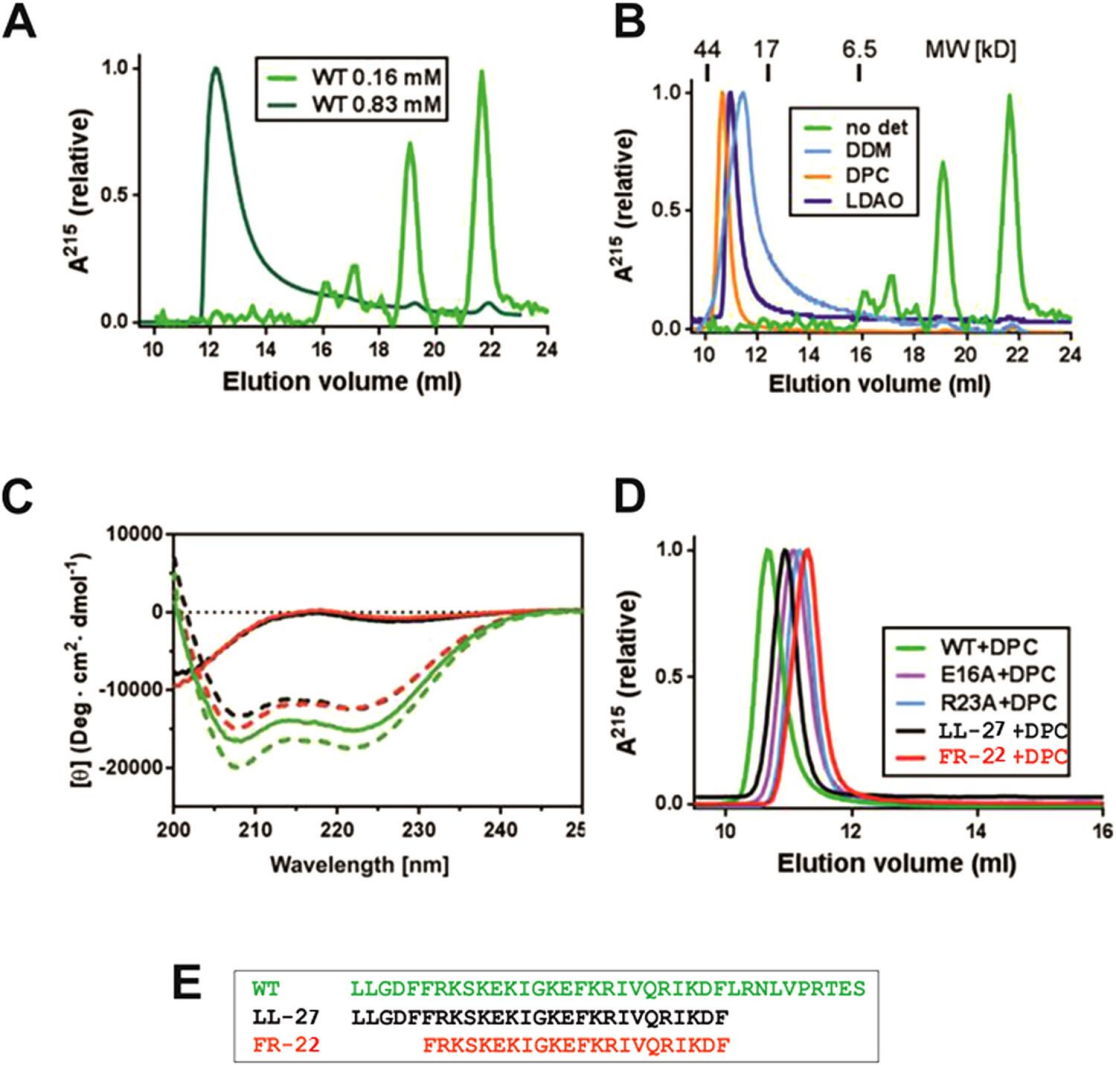
Biochemical analysis of LL-37 oligomeric peptide states induced by detergents. (**A**) Size exclusion chromatography (SEC) of LL-37 samples at 0.16 (light green) and 0.83 mM (dark green) shows a concentration dependent oligomerization. (**B**) SEC of LL-37 in the presence of the detergents DDM, DPC and LDAO (light and dark blue and orange) show a significant shift in the molecular weight to around 30 kD. (**C**) Circular dichroism spectra of LL-37 show structural maintenance of LL-37 in the presence of detergents and structural remodeling of the truncated variants through detergents. (**D**) SEC of LL-37 variants in the presence of DPC. In green the wildtype LL-37 peptide, the E16A mutant in magenta, the R23A mutant in blue, the LL-27 and FR-22 variants are in black and red, respectively. (**E**) Sequences of the truncated LL-37 construct LL-28 and FR-23 used for biochemical analysis.

To further verify the oligomeric states obtained by crystallography using complementary methods we used size exclusion chromatography (SEC), circular dichroism (CD), and analytical ultracentrifugation (AUC). In SEC, LL-37 shows a concentration dependent oligomerization behavior. At low concentrations of 0.16 mM the dominating species is monomeric, while at concentrations of 0.83 mM the peptide showed retention times in the range of a tetramer or hexamer (see Fig. 6A). By contrast, SEC experiments performed in the presence of the detergents LDAO, DPC and DDM yielded molecular weights of tetramers or hexamers even at the low peptide concentrations of 0.16 mM (see Fig. 6B). Circular dichroism spectra of LL-37 shows the same secondary structure content in the absence and presence of detergents (see Fig. 6D). LL-37 in DDM has also been used for analytical ultracentrifugation (AUC) at a detergent concentration slightly higher than the critical micellar concentration. The oligomerization state induced by DDM returned a molecular weight of ~28 kD which indicates tetra- or hexamerization of the peptide (data not shown).

### The fold and activity of truncated LL-37 variants is restored by detergents

In order to test the structure-based activity of LL-37 mutants, and to narrow down the peptide length towards a core structure element still harboring activity (marked in red), we developed two truncated variants termed LL-27 (C-terminal truncation) and FR-22 (N- and C-terminal truncation; see Fig. 6E). First we tested the folding of the peptides and it turned out that both are unfolded in the absence of detergents but fully folded in the presence of DPC (see Fig. 6C). Next we analyzed their oligomeric states and observed that the peptides were monomeric in the absence of detergents but showed essentially the same molecular weight in SEC as LL-37 in the presence of detergents (see Fig. 6D). Finally, both peptides and wildtype LL-37 were tested for their antimicrobial activity against *E. coli*. First we tested the wildtype peptide in the presence of detergent and to our surprise the activity of LL-37 was enhanced from 6 to 3 μg/ml in the presence of LDAO (see Table SII). The truncated peptides were essentially inactive in bacterial killing with MICs > 50 μg/ml but their activity could be restored to MIC values of 6 μg/ml (in the case of LL-28) in the presence of 0.005% of LDAO or 0.01% DDM (see Table SII).

## Discussion

Antimicrobial peptides such as the membrane-targeting LL-37 are considered to be one potential source for the development of new alternative antibiotics in the future. In spite of their co-evolution with bacteria, many of these peptides have retained activity against multidrug resistant strains including *Staphylococcus aureus* and *Acinetobacter baumanii* ^15^. Their success in killing a wide spectrum of Gram-positive and -negative bacteria is based on high physiological concentrations providing constant protection^20,21^, along with often unspecific targeting mechanisms of bacterial cell envelopes^5,6^. One important prerequisite to bring new AMPs towards clinical trials is furthering our understanding of their individual antimicrobial mechanisms. We believe that these mechanisms are diverse and more sophisticated than outlined by the three fundamental models (barrel stave, toroidal and carpet)^2,5,6^.

Many AMPs interacting with membranes can switch between several structural states, one of which is in solution while additional states may be adopted in the presence of lipids/membranes. Changes between those states can have an impact on the 3D structure but also on the oligomerization state of the peptides. The structural transition of AMPs from one state to another, although biologically interesting, has so far rarely been attempted at atomic resolution. Human cathelicidin is one of the best-studied antimicrobial peptides, but the full understanding of its antimicrobial activity based on membrane perturbation is hampered by the difficulty to study structural changes at the membrane interface. Here, we describe our investigation of the mechanism by which LL-37 targets membranes using structural and biochemical techniques. First we determined the dimeric and amphipathic structure in the absence of detergents. Following the procedures developed for membrane protein crystallization various detergents as membrane-mimicking factors were tested for the co-crystallization with LL-37. The presence of LDAO and DPC detergents induced a significant structural rearrangement all through the molecule yielding co-crystal structures of a new molecular state. Along with the rearrangement induced by detergents a hydrophobic scaffold spanning the dimer interface was induced accommodating four detergent molecules per peptide dimer (see Fig. S3). To our knowledge, this is the first time that two structurally different states and discrete detergent binding sites in membrane-interacting peptides are reported. Their analysis unravels an unexpected plasticity for this particular human AMP and the structural transition may also be due to the importance of the peptide to discriminate between host and pathogen membranes (see Figs 2 and S3 and the following paragraphs).

Previously, LL-37 was studied by NMR but the structural information gained differs considerably from the observed crystals structures. In the presence of SDS or DPC micelles, the NMR assignment resulted in monomeric peptide models of a strongly bent (using SDS) or a helix-break-helix (using DPC – no PDB data are available for direct comparison) conformation (see Fig. S6)^12,22^. NMR data using DPC micelles confirmed the detergent induced structural changes at the N-terminus of LL-37_DPC-2_ with a random coil rather than an α-helical structure. Furthermore, the structural disorder detected in the C-terminus of LL-37_DPC-2_ is in good agreement with the NMR data (see Fig. 2)^12^. Finally, detergent interactions reported for LL-37_LDAO-2_ are in line with NMR studies using deuterated DPC: here NOESY analysis of spectra using perdeuterated DPC suggests that aromatic residues Phe5, Phe6, Phe17 and Phe27 are directly involved in detergent interactions. These observations highlight the importance of aromatic residues in antimicrobial peptides for detergent or lipid binding, respectively, in analogy to previously reported outer and inner membrane protein structures (see Fig. S3 for a model of LL-37 binding PE)^12,22,23^. In summary, the combined structural information confirms discrete detergent binding sites with hydrophobic alkyl chain conformations likely resembling lipids conformations *in vitro* and *in vivo* (see Fig. S3C). Therefore it is tempting to speculate that the structure of LL-37_LDAO-2_ represents the first state of peptide-membrane interactions after the extraction of lipid molecules from the membrane and their incorporation into peptide-lipid complexes (see Fig. S3). Using the simple mechanistic pictures (toroidal, carpet-like and barrel stave) describing peptide-membrane interactions, LL-37 shows mechanistic features of both, the toroidal-like (by interacting with lipids) and the carpet-like mechanism (by extracting lipids from the membrane). However, further studies addressing the exact mechanistic sequence have to be done on membrane and in bacterial cells or isolated cell envelopes.

Recent studies on AMPs suggested their potential supramolecular organization on membranes at physiological concentrations *in vivo* and *in vitro* as an intermediate step towards membrane perturbation and bacterial killing. When we analyzed the crystallographic packing of LL-37_LDAO-2_ and LL-37_DPC-2_ in this context it turned out that one-dimensional chains are formed by hydrophobic head-to-tail interactions in both crystal forms (see Figs 4 and S4A). The building block of these chains is the LL-37 tetramer, which would be in line with the data from size exclusion chromatography or cross-linking results (see Fig. 6). Also in line with our observation of fiber-like structures are reports demonstrating the formation of one-dimensional peptide chains by the designer peptide LAH4, BTD-2 and LL-37 on vesicles pointing towards a similar mode of self-organization patterning^24–27^. However, these observations are only indirect, using imaging techniques, while our structures are at the atomic level and show the fiber assembly. To confirm the formation of these fiber-like structures we used gold-labelled LL-37 and showed elongated structures on vesicle membranes using electron microscopy (see Fig. 5). We assume that these structures can also form *in vivo* (see Fig. S7), and these surface-exposed nests or grooves (see previous paragraph) for the extraction of lipids from membranes may be developed. Lipid extraction from membranes may also facilitate the subsequent voltage-driven membrane insertion of the peptides to form channels/pores perforating the inner membranes (see Figs S7 and S8). Based on the data presented and integrating results from the recent literature we present a model how LL-37 may interact with and kill Gram-negative bacteria (see Fig. S8). In this model LL-37 initially targets and extracts LPS of the outer membrane to form holes which may allow for the diffusion of peptides into the periplasmic space. LL-37 may further interact with the inner membrane after local extraction of lipids, the formation of the membrane state (LL-37_LDAO-2_) and followed by oligomerization and fiber formation on the membrane.

Cathelicidins are tissue-specifically expressed in mammals at higher physiological concentrations but appear to be of low toxicity for eukaryotic cells, and their functional properties allow them to discriminate between prokaryotic and eukaryotic membranes. To investigate the peptide-lipid specificity in more detail, the affinity of LL-37 towards a set of representative lipids was examined. Both pro- and eukaryotic lipids were tested, and a clear preference of LL-37 towards major bacterial lipids (PE, PS, PG and CL) could be demonstrated. Eukaryotic membranes are rich in PS, which is also recognized by LL-37. However, under non-apoptotic condition PS is normally facing the inner leaflet of the membrane and is therefore not accessible to the peptide^28^. Interestingly, the overall charge of the lipid head group does not exclusively determine the binding probability as previously reported. The lipid head group structure appears to be the second determinant for affinity as demonstrated for PI (charge -1) and PI-4 (charge -3), which do not show binding in spite of their negative charges (see Fig. 5A). The discrimination of eukaryotic PC and bacterial PE is particularly revealing for the mechanism, as the head group of PC is not recognized by LL-37. This implies that bulkiness near the phosphate group could be an important selection criterion for interactions by shielding the negative charge, similar to the bulky PI head group (see Fig. 5B). This model is in line with the mechanism of a hydrophobic nest, which offers space for the binding of two lipid molecules in close proximity and an essential Arg23 residue that could select for the lipid charges. Taken together, these results are important to understand how LL-37 may selectively target bacterial but not eukaryotic membranes indicating the importance of single conserved residues (Arg23) in lipid recognition.

What can we learn from the repertoire of structures presented here with respect to the structure-based design of LL-37 constructs for clinical studies? There are three principal requirements for the logical design of LL-37 based mutants: (1) the length of the peptide has to be reduced due to economical and purity considerations; (2) the activity of the peptide against bacterial cells should be in the range of wildtype and (3) the affinity towards eukaryotic cells should be low. Based on LL-37_LDAO-2_, we designed two peptides that essentially contained the helical core structure elements (see Figs 2, 6C). These peptides of 27 and 22 residue lengths were unfolded and presented less activity in the absence of detergents, but then recovered activity by folding in the presence of the detergents. Both mutants contained all residues essential to form the nest-like structures and contain the Arg23 residue, which may be important for lipid selection. Detergents are known for their properties to support antimicrobial activity in an additive and synergistic way. The detergents added in our study may be important for the stabilization of the central dimerization interface (LDAO2 and LDAO3 positions), and their type and concentration could be varied to yield even more pronounced effects. Based on these results the rational development towards optimized peptides and peptide/detergent ratios could be tested. Improved peptides are valuable for clinical groups aiming on the development of new peptide-based antibiotics.

## Methods

### Oligomerization studies of wildtype and mutant LL-37 using size exclusion chromatography (SEC)

The oligomeric state of the peptide in the absence and presence of detergents was characterized on Superdex75 10/300 GL column (GE Healthcare) at peptide concentrations of 0.7 (0.16 mM) or 3.7 mg/ml (0.83 mM) respectively. The column was equilibrated using buffer A containing 100 mM NaCl, 10 mM NaHCO_3_, 5 mM sodium phosphate, pH 6.8, for the detergent free sample. SEC in the presence of detergents was performed at 0.7 mg/ml (0.16 mM) of LL-37 in buffer A plus the following detergent concentrations: 0.2% LDAO (8.7 mM), 0.05% DDM (0.98 mM), 0.2% DPC (5.6 mM). Peptides were incubated with buffer A and buffer-detergent solutions for 30 minutes before 200 μl samples were loaded onto the column. The flow rate of the column was adjusted to 0.5 ml/min and all runs were performed at room temperature. The peptide retention time was monitored at 215 nm due to the absence of Trp and Tyr residues.

### Characterization of peptides by circular dichroism (CD)

Wildtype and mutant peptides applied to SEC were also used for the secondary structure determination using a CD JASCO J-810 spectrophotometer (Jasco Spectroscopic Co. Ltd., Hachioji City, Japan). Peptides were analyzed at 20 °C and a scanning speed of 20 nm/min with a band width of 1 nm. All spectra were recorded at 0.2 nm resolution and outputs were reported as differences in molar absorption. The samples were measured in quartz precision cuvettes with a path length of 2 mm. The analysis of CD spectra for the estimation of secondary structure contents was performed using the programs SELCON3, CDSSTR and CONTIN from the CDPro software package^29^.

### Labelling of LL-37 with nanogold and preparation of lipid vesicles for cryo-EM

For the labeling of LL-37 with 1.4 nm nanogold particles (Nanoprobes, Inc), 200 µM of the S37C peptide were reduced using 5 mM of Dithiothreitol (DTT) in the presence of 5 mM EDTA and 100 mM sodium phosphate, pH 7, at room temperature for 1 hour while shaking. The excess of DTT was removed using a 3 kD-cutoff ultrafiltration cell (Amicon, Millipore). Nanogold at 6 nmol concentration was incubated with the peptide at room temperature for 2 hours while shaking. To remove the excess of nanogold, the mixture was loaded onto a Superdex 75 10/300 GL column (GE Healthcare) with 150 mM NaCl and 20 mM sodium phosphate, pH 7. The fractions containing the labeled peptide were concentrated using a 3 kD-cutoff (Amicon, Millipore) ultrafiltration device. The DOPC:DOPG (3:7) SUVs (small unilamellar vesicles) were prepared using a mini-extruder (Avanti Polar Lipids, Inc). Their size and homogeneity was checked using dynamic light scattering (DLS). DOPC:DOPG vesicles were treated with nanogold labeled LL-37 (ratio of peptide to lipid 1:250).

*E. coli* K12 cells were grown at 37 °C at 200 rpm up to an OD of 0.6, and labeled LL-37 nanogold was added to 1 ml culture to reach a final concentration of 18 µM (80 µg/ml). The cultures were further incubated at 37 °C while shaking at 300 rpm (Eppendorf Thermomixer, Hamburg, Germany). After 80 minutes, 200 μl of the sample were taken and cells were pelleted at 3000*g for 10 min at 4 °C. The cell pellet was resuspended using 15 μl of culture solution in order to concentrate the sample and reach a sufficiently high cell density for the visualization in the microscope.

### Lipid affinity assays of LL-37 using lipid strip technology

The lipid affinity of LL-37 was assessed using peptide-lipid overlay assays of commercially available lipid strips blotted with 100 pmol of biologically relevant lipids (Echelon Biosciences, Salt Lake City, UT), following the manufacturer’s instructions. Lipid strips were blocked with 1% fat-free milk in TBS (150 mM NaCl, 50 mM Tris-HCl, pH 7.6) for 1 h at room temperature and then incubated with LL-37 (0.04 mg/ml) dissolved in 10 mM NaHCO_3_, 100 mM NaCl, 5 mM sodium phosphate, pH 8, plus 1% fat-free milk in TBS for 1 h at room temperature. The lipid strips were first washed with TBS buffer three times, and then incubated overnight at 4 °C in a solution of TBS containing 1% fat free milk and the anti-LL-37 HRP-labelled antibody (Innovagen, Sweden) in a 1:2000 dilution. After being washed three times with TBS, the peptide was detected by chemoluminescence using the super-signal western femto maximum sensitivity substrate kit (Thermo Scientific, Pierce Biotechnology, Inc).

### Crystallization and crystallographic studies of LL-37

The peptide was used for the sitting drop crystallization attempts at concentrations of 20–40 mg/ml. Crystallization of the peptide alone was conducted using peptide-detergent solutions of 0.5% in SEC buffer containing 2 mM sodium phosphate buffer, pH 6.8, using the commercial screens from Jena Bioscience, Qiagen, and Molecular dimensions. Sitting drops of 400 + 400 nl (peptide + reservoir) were prepared by a Mosquito robot (TTP Labtech) and the progress of the crystal formation was monitored using the Formulatrix Rock imaging system. Crystals were mounted from crystallization drops and data were collected at the synchrotron sources SLS (Swiss Light Source, Villigen, Switzerland - beamline PX10) and ALBA (Barcelona, Spain - beamline BL13-XALOC; structures in the presence of detergents). Data were recorded on the Pilatus detector 6 M (Dectris) at 100 K with beam attenuation of 20–50% at beamline PX10. Data were processed using the XDS/XSCALE program package^30^. The structure of the dimer of LL-37 in solution (termed LL-372) was solved by direct methods using the Arcimboldo program and utilizing an α-helix as starting model^31–34^. The structure of monomeric LL-37 crystallized in DPC (termed LL-37_DPC-1_) was solved by molecular replacement (MR) using 2O6N as the search model^35^. The refined high-resolution structure of this monomer was further successfully employed as a search model to solve the dimer structures crystallized in LDAO and DPC (termed LL-37_LDAO-2_ and LL-37_DPC-2_). The structures were refined by Refmac^36^, Phenix^37^, and BUSTER^38^, and manually modelled using the COOT program package^39^. Based on F_O_ − F_C_ difference maps, detergent molecules were added and refined together with the peptide model. The geometry of the structures was validated using the Molprobity server (http://molprobity.biochem.duke.edu/). All refinement and model statistics are given in Table S1.

### Data Availability

The structures have been deposited at the PDB database under the following numbers: 5NMN - LL-37_DPC-1_, 5NNM - LL-27, 5NNK LL-37_LDAO-2_, 5NNT - LL37_DPC-2_.

## Electronic supplementary material

Supplementary information
