# Supplementary material for: Structural remodeling and oligomerization of human cathelicidin on membranes suggest fibril-like structures as active species
Source: Sci Rep. 2017 Nov 13;7:15371. doi: 10.1038/s41598-017-14206-1 (PMC5684418; doi:10.1038/s41598-017-14206-1)
Supplement: Supplementary file 1 — Supplementary information [file 41598_2017_14206_MOESM1_ESM.pdf]

Supplementary material to the paper:

Structural remodeling and oligomerization of human cathelicidin on membranes suggest fibril-like structures as active species

by Enea Sancho-Vaello, Patrice François, Eve-Julie Bonetti, Hauke Lilie, Sebastian Finger, Fernando Gil-Ortiz, David Gil-Carton, Kornelius Zeth

Peptides, lipids and detergents

All peptides used in our study were chemically synthesized and purchased from Peptide2.0(www.peptide20.com), Proteogenix (www.ProteoGenix.fr) or Genecust (www.genecust.com)at purities of higher than 95%. Detergents used in this study are N-octyl-β-D-glucoside(Apollo Scientific limited), N-Dodecyl-β-D-maltoside (Apollo Scientific limited), N-dodecylphosphocholine(Affymetrix) and N-dodecyl-N,N-dimethylamine-N-oxide(Affymetrix). The lipids 1,2-dioleoyl-sn-glycero-3-phosphocholine (DOPC), 1,2-dioleoyl-snglycero-3-phosphoglycerol(DOPG) and cardiolipin (CL) were purchased from Avanti PolarLipids, Inc. (Alabaster, AL, USA).

Analytical ultracentrifugation (AUC)

Equilibrium sedimentation measurements were conducted using a Beckman analyticalultracentrifuge XL-I equipped with an An50Ti rotor and double sector cells. Centrifugationruns were performed at 18.000 rpm and 20°C; data were collected at a wavelength of 230 nmusing a concentration of 0.33 mg/ml (0.07 mM) in 50 mM phosphate buffer, pH 7.2. In thepresence of 0.04% DDM (0.78 mM) the impact of the detergent on the sedimentationinfluence of LL-37 was removed using the approach of gravitational transparency and thedensity of the buffer was adjusted to the density of hydrated DDM micelles. Densityadjustment was achieved by variation of sucrose concentrations as coagulant in the buffer(see Fig. 4D). The radial distribution of LL-37 at sedimentation equilibrium in the respective bufferwas linearized according to the Lamm equation [1]and the slope dlnc/dr2 with c representingthe peptide concentration at the radial position r was determined experimentally. This slopewas plotted against the density of the buffer and

interpolated to the density of hydrated DDMmicelles (1.1167 g/ml). The slope at this density was used to calculate the molecular mass ofLL-37 according to the following equation:

$$M(1-v f) = 2RT/k2 * d\ln c/dr^2$$

where v is the partialspecific volume of LL-37, R the universal gas constant, T the absolute temperature, *k* theangular velocity, c the peptide concentration, and r the radial distance. In the absence of thespecific information on hydration of LL-37 in DDM micelles, a partial specific volume of v =0.757 cm3/ml which is the average between the partial specific volume of a fully hydratedprotein in sucrose buffer [2] and the estimated density of a non-hydrated protein[3] wasassumed. The entire method of analytical ultracentrifugation applied to membrane proteinsand their data analysis is described in [4].

ATR-IR spectroscopy of LL-37 in lipid vesicles

Temperature dependent ATR-Fourier transform IR spectroscopy data were recorded with aspectral resolution of 4 cm-1using a Bruker Tensor 27 spectrophotometer equipped with anN_2-cooled MCT detector and a BioATR II unit (Bruker Optics, Ettlingen, Germany). Thetemperature was adjusted by a computer-controlled circulating water bath (Haake C25PPhoenix II, Karlsruhe, Germany). Each spectrum shown represents the average of 256individual wavelength scans. The final absorbance spectra for each temperature werecalculated by -lg ($I_{sample}/I_{reference}$). The spectrum of pure water at each temperature was used asa reference. The sample prepared using extruder technology contained 100 nm vesicles, asolution of LL-37, and the lipid/peptide mixtures. Defined aliquots of peptide and vesiclesolutions were mixed above the lipid phase transition temperature directly on the crystalsurface of the cell to obtain a charge ratio of one and a lipid concentration of 1 mM. Beforerecording spectra, one heating and cooling scan was performed to ensure equilibration ofthesystem. Spectra were recorded in 2°C intervals after temperature equilibration of±0.1°C for15 min was maintained. The temperature was determined inside the cover plate of the sampleholder by a Pt100 resistor (Omega Newport, Deckenpfronn, Germany).

For further data analysis absorbance spectra were shifted to a zero baseline in aspectral region where no vibrational peak occurred. To determine the position of the CH_2vibrational bands in a certain wavenumber interval by calculating the second derivative of thespectra, we used the ‘peak picking’ function included in the Bruker OPUS software. Forcomparison of peak position and intensity ratios the spectra were normalized to the amide Iintensity of 0.1. Experimental intensities of the amide I band varied between 0.1 and 0.2depending on sedimentation of vesicles in solution.

References

1. Chervenka, C. *A manual of methods for the analytical ultracentrifuge*. (Beckman Instruments Inc., 1970).

2. Rickwood, D. *Centrifugation. A practical approach.* (IRL Press, 1984).

3. Ralston, G. *Introduction to Analytical Ultracentrifugation*. (1993).

4. Lustig, A., Engel, A., Tsiotis, G., Landau, E. M. & Baschong, W. Molecular weight determination of membrane proteins by sedimentation equilibrium at the sucrose or nycodenz-adjusted density of the hydrated detergent micelle. *Biochim. Biophys. Acta* **1464,** 199–206 (2000).

5. Aisenbrey, C. & Bechinger, B. Molecular packing of amphipathic peptides on the surface of lipid membranes. *Langmuir* **30,** 10374–83 (2014).

Supplementary figuresand figure captions

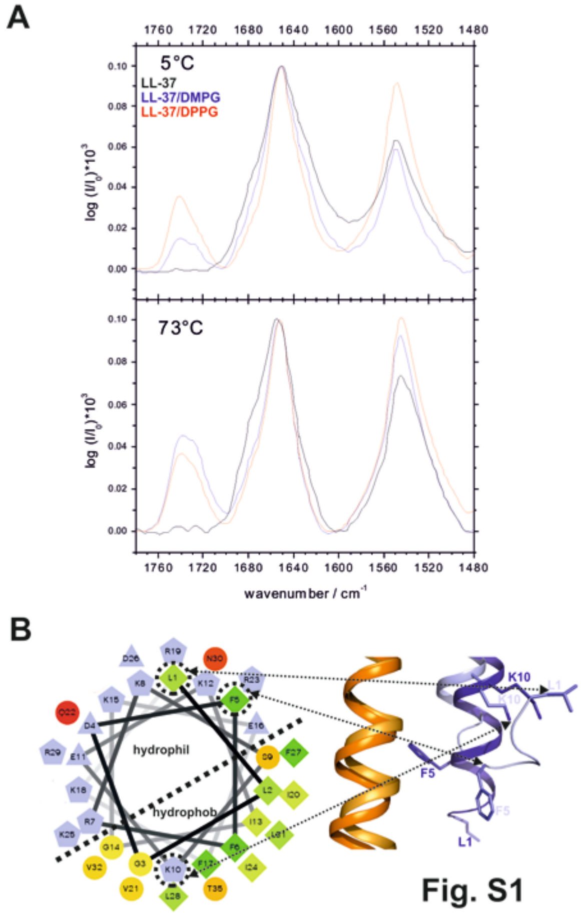

Figure S1 - Structural stability of and helical wheel representation of LL-37.(A) Thermal stability assay of LL-37 using FTIR at 5ºC (top) and 73ºC (bottom). LL-37samples were analyzed in solution and in the presence of lipid vesicles (DMPG and DPPG)and both samples demonstrated clear secondary structure maintenance up to a temperature of73 degrees. **(B)** Helical wheel representation of the LL-37 sequence demonstrates theamphipathic nature of the peptide under the assumption that the peptide assumes a α-helicalstructure. Notably, residues Leu1, Phe5 and Lys10 marked by dashed circles can beconsidered outliers (in the solution structure) but their deviation can be explained by thestructural transition from an α -helical conformation in LL-37 towards random coil in the LL-37$_{LDAO-2}$ structure.

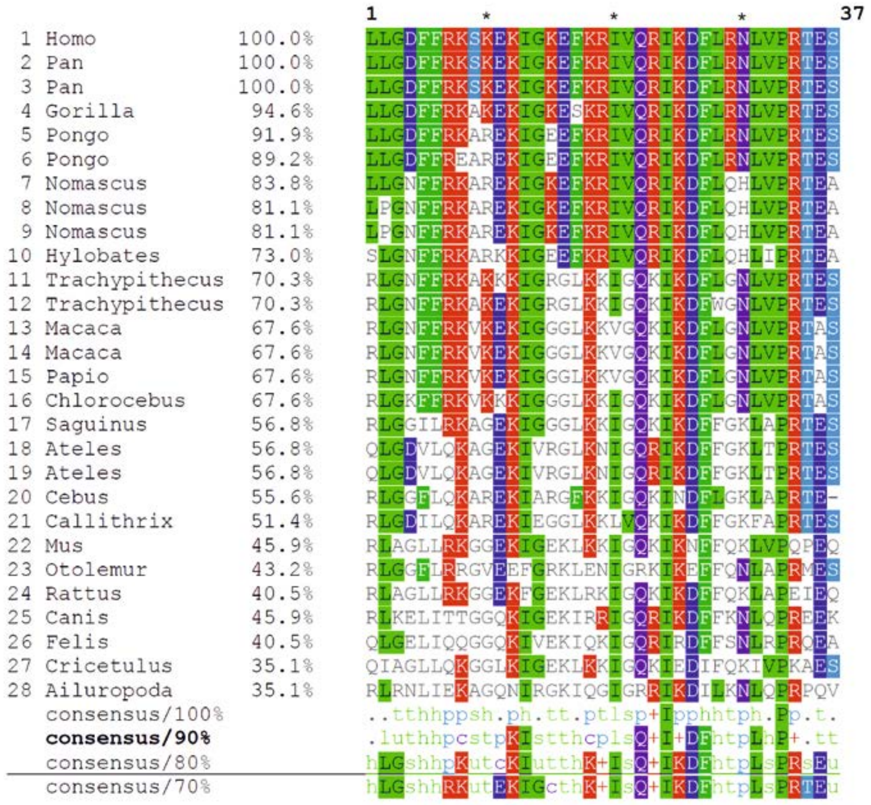

Fig. S2

Figure S2 - Sequence alignment of cathelicidin sequences. Alignment of currently available cathelicidin sequences. Their sequence identity relative tohuman LL-37 (homo) is given. The alignment was generated using the fasta33 program(http://www.ebi.ac.uk/Tools/sss/fasta/).

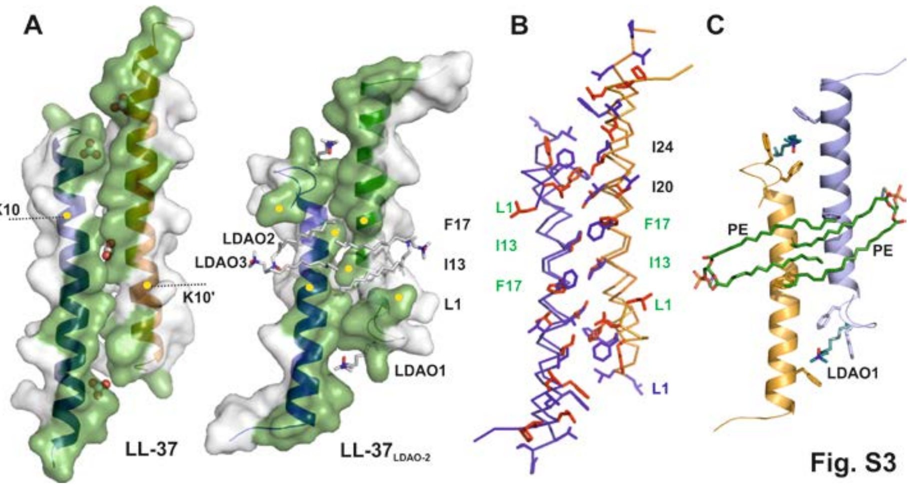

Figure S3 - Surface representation of LL-37 LDAO-2 indicates an interaction of the peptidewith alkyl chains suggesting lipid binding. (A) The comparison of LL-37 structures using surface representations of LL-37_2 andLL-37_{LDAO-2} demonstrates significant changesin surface properties. The location of the outlier residue Lys10 breaking the hydrophobicpattern identified by the helical wheel plot (see Fig. S1B) is marked, LDAO detergentmolecules (LDAO-1, LDAO-2 and LDAO-3) are shown in stick representation in LL-37_{LDAO-2}.Hydrophobic residues involved in detergent binding at the center of the structure are markedwith yellow circles. **(B)** Superposition of LL-37_2 and LL-37_{LDAO-2} structures shown as backboneribbons with hydrophobic residues forming the central hydrophobic pockets marked as sticksand are numbered according to the sequence. Apart from the significant structuralrearrangement of N- and C-terminus also residues central to the structure of LL-37_{LDAO-2}(Leu1, Ile13 and Phe17) show changes in their conformations in response to detergentinteractions. **(C)** Hydrophobic tails of LDAO molecules may indicate the localization ofphospholipid binding sites if the peptide was exposed to membranes. Superposition PErelative to LDAO2 and LDAO3 using the tail alignment. Detergent molecules are omitted forclarity.

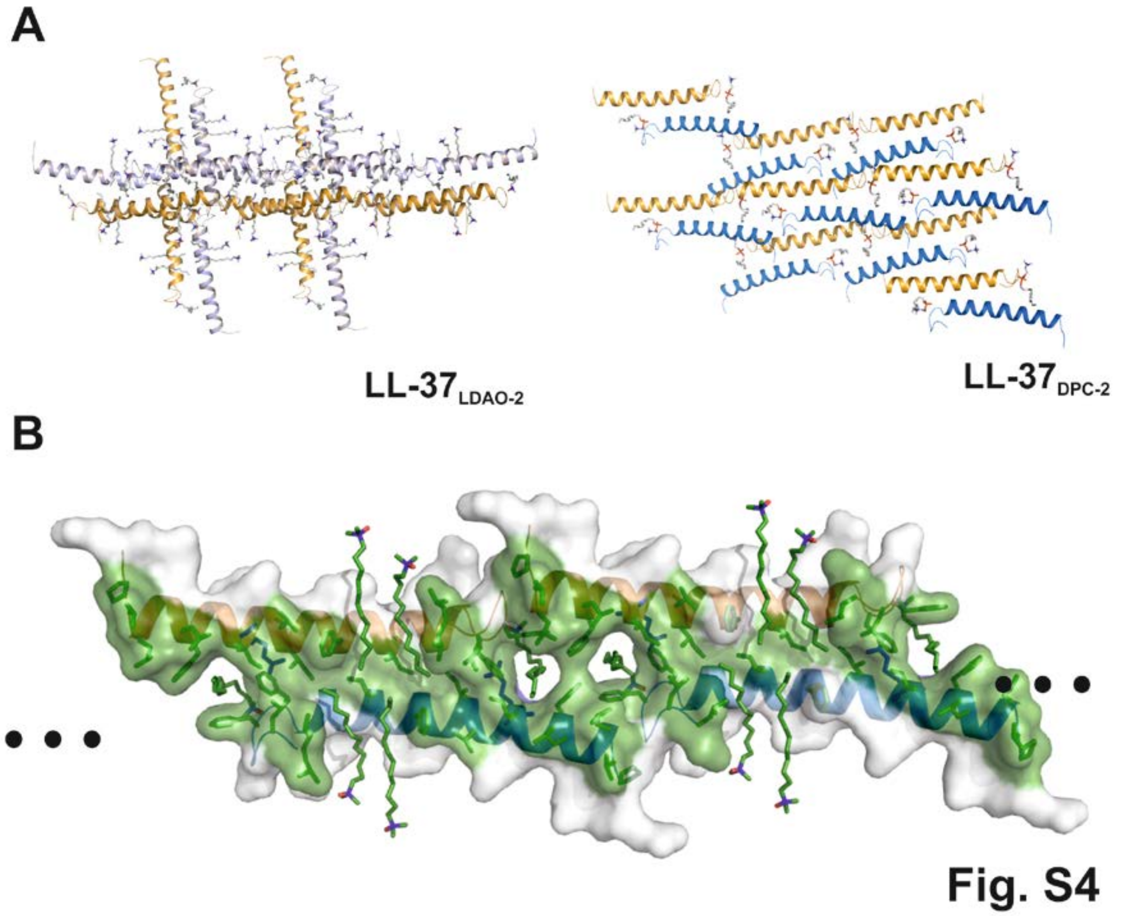

Figure S4 - Comparison of LL-37$_{LDAO-2}$ and LL-37$_{DPC-2}$ crystal packing and theformation of 1D chains in the crystal lattice. (A) Crystal packing of LL-37$_{LDAO-2}$ and LL-37$_{DPC-2}$ are displayed (see alsotable SI). **(B)** In spite of the crystallographic differences, 1D chains are identical in bothcrystals and the surface of one tetramer formed by head-to-tail interactions is shown.Hydrophobic residues forming an extended hydrophobic patch are marked in green.

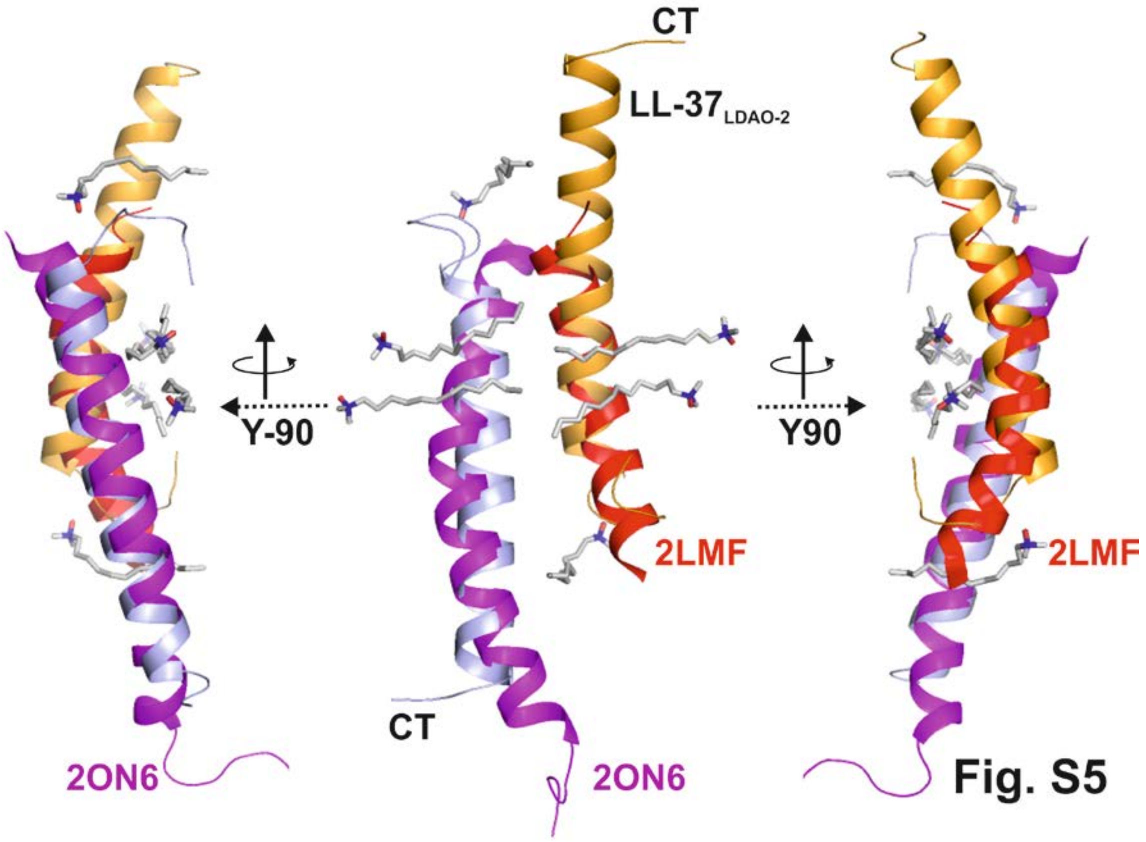

Figure S5 - Comparison of LL-37 crystal and NMR structures. Structural superposition of LL-37_{LDAO-2} shown in light purple and orange and NMR structuresdetermined in the presence of SDS (LL-37 full length structure; 2ON6) and DPC (truncatedversion of LL-37; 2LMF).

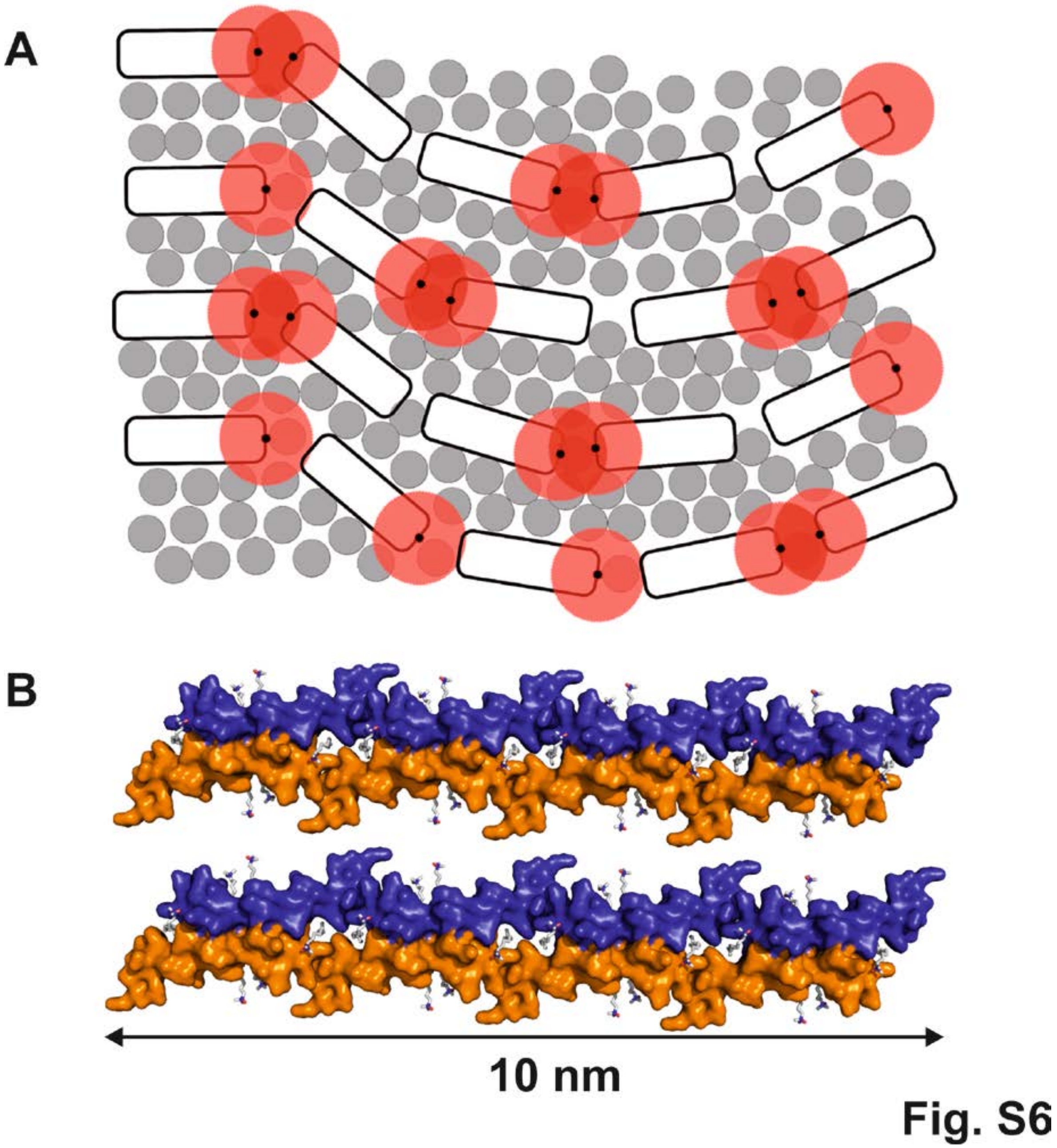

Figure S6. (A) Models for the supramolecular structure formation of LL-37 and LAH4.**(B)** Model for the head-to-tail oligomerization of the synthetic LAH4 peptide[5] in comparison to the 1D association of LL-37 in chains observed in the crystal packing.

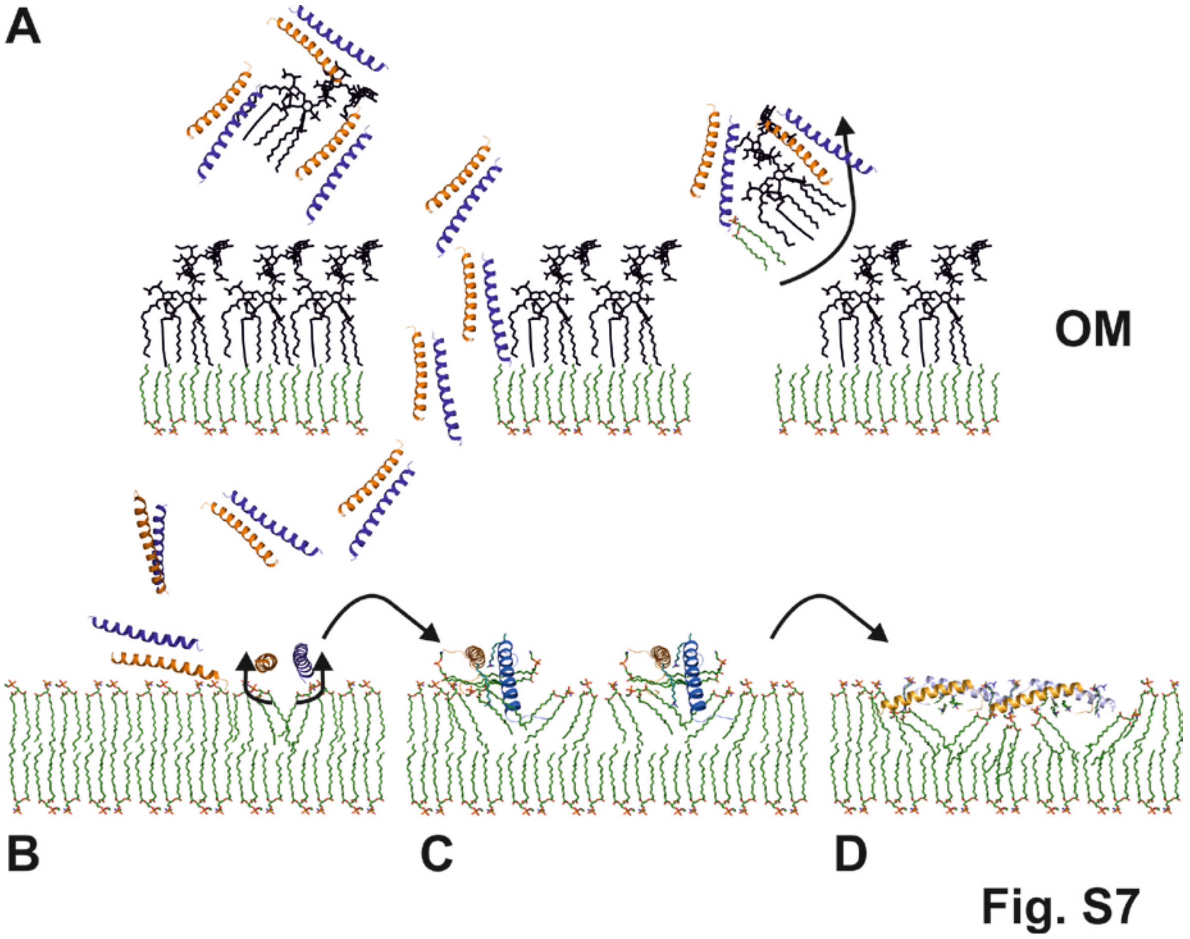

Figure S7- Proposed model of LL-37 interactions with the bacterial cell wall**(A)** Based on the results presented in this paper and the recent literature and we propose amodel of LL-37 interactions with the cell wall of *E. coli*. Initial interactions are driven byelectrostatic attraction with the outer membrane occur via LL-37/LPS complexes which lead to thedisplacement of patches from the outer membrane. **(B)** LL-37 can interact with the innermembrane based on electrostatic interactions with lipids such as PE. **(C)** Lipids may be extracted from the membrane leading to form an activated peptide conformation LL-$37_{LDAO-2}$and further the formation of supramolecular structures **(D).**

Table 1

Data collection and refinement statistics

	LL37	$LL37_{LDAO}$	$LL37_{DPC-1}$	$LL37_{DPC-2}$
**Data collection**				
Space group	$P3_121$	$I4_122$	C2	$P2_12_12_1$
Cell dimensions				
a, *b*, *c* (Å)	44.58, 44.58, 87.84	43.31, 43.31, 109.82	60.38, 17.83, 34.03	35.29, 43.79, 54.50
α, β, γ (°)	90 , 90, 120	90	90, 105.15, 90	90
Resolution (Å)	38-1.9<br>(2.01-1.9)	21-1.80<br>(1.91-1.85)	30-0.95<br>(1.01-0.95)	50-2.2<br>(2.32-2.2)
R_{sym} or R_{merge}	0.10 (0.34)	0.06 (0.59)	0.04 (1.09)	0.13 (0.85)
CC* in outermost shell	89.2	31.4	91.7	80.3
$I / \sigma I$	7.75 (2.51)	15.3 (1.3)	16.18 (1.36)	8.1 (2.31)
Completeness (%)	99.2 (98.3)	94.0 (69.7)	92.6 (79.2)	98.7 (95.3)
Redundancy	3.5 (3.5)	9.0 (2.9)	4.8 (3.8)	6.0 (5.4)
**Refinement**				
Program	REFMAC	PHENIX	PHENIX	PHENIX
Resolution (Å)	38-1.9<br>(1.95-1.90)	21-1.8<br>(2.06-1.8)	30-0.95<br>(1.01-0.95)	29-2.2<br>(2.8-2.2)
No. reflections	7055	8560	20639	4433
R_{work} / R_{free}	0.23/0.25 (0.32/0.39)	0.25/0.26 (0.42/0.51)	0.15/0.15<br>(0.37/0.37)	0.26/0.28<br>(0.32/0.37)
No. atoms				
Protein	600	766	374	635
Water	25	1	15	10
Carbonate	4			
B-factors				
Protein	50.8	40.2	29.5	32.1
Water	55.2		37.8	
R.m.s. deviations				
Bond lengths (Å)	0.04	0.006	0.001	0.003
Bond angles (°)	2.90	1.32	0.92	0.74
**Ramachandran statistics**				
Residues in favoured region No (%)	100	100	100	100
Residues in allowed region No (%)	100	100	100	100
Residues in outlier region No (%)	0	0	0	0
**PDB-entry**				
**Crystallization conditions**				

*Values in parentheses are for highest-resolution shell.

Table SII:

Antimicrobial activity testing of LL-37 and two truncated variants applied to *E. coli* K12 in the absence and presence of detergents.

	MIC (µg/ml) *E. coli* K12
**WT**	6.25
**LL-27**	> 400
**FR-22**	> 400
**WT + 0.005% LDAO**	3.12
**LL-28 + 0.005% LDAO**	6.25
**FR-23 + 0.005% LDAO**	25
**WT + 0.01% DDM**	6.25
**LL-27 + 0.01% DDM**	6.25
**FR-22 + 0.01% DDM**	25
